# Discrepancy Rates in Acute Abdominal CT: An Audit of In-House and Remote Reporting Compared to Intraoperative Laparoscopic and Laparotomy Findings

**DOI:** 10.7759/cureus.73509

**Published:** 2024-11-12

**Authors:** Ishtar A Redman, Georgios Ntampakis, Amin Alamin, Anand Mohan, Kyriakos Bananis, Panagiotis Drymousis

**Affiliations:** 1 Orthopedics and Trauma, University College London Hospitals NHS Foundation Trust, London, GBR; 2 General Surgery, Ealing Hospital, London North West University Healthcare, NHS Foundation Trust, London, GBR

**Keywords:** acute abdomen, ct discrepancies, general surgery, in-house reporting, intraoperative findings, laparoscopy, laparotomy, off-site reporting, teleradiology

## Abstract

Introduction: Non-traumatic abdominal pain is a common emergency presentation frequently managed by general surgeons. Abdominopelvic computed tomography (CT) scans are the most popular imaging modality in this context. In many hospitals, the rising demand for urgent and emergent scans out-of-hours has necessitated the outsourcing of this service to teleradiology companies, whereby reports are generated at sites remote from the image acquisition. The primary aim of this study was to determine the discrepancy rates of preoperative CT imaging by source (teleradiology compared to in-house).

Methods: This was a retrospective monocentric study conducted at a busy district general hospital over a seven-month period. Patient demographic data, operative notes, and radiology reports (by source) were collated for all patients aged ≥16 years presenting with atraumatic abdominal pain who underwent abdominopelvic CT with subsequent surgical intervention (laparoscopy and/or laparotomy).

Results: Seventy-one patients were identified by initial screening, and 10 patients (11 scans) met the criteria for a "major" discrepancy. Overall discrepancy rates were calculated at 5.6% for scans reported off-site compared to 9.9% for reports generated by in-house radiologists.

Conclusion: This study demonstrated lower discrepancy rates in scans reported remotely and can be used as the catalyst for improving aspects of in-house CT reporting.

## Introduction

Non-traumatic abdominal pain is a common presentation in accidents and emergencies with a broad differential, ranging from relatively benign conditions such as gastritis to more serious, hollow viscous perforation [1]. Patients with a "surgical abdomen" often provide a history of sudden onset severe abdominal symptoms indicative of a possible life-threatening intra-abdominal pathology, with most cases requiring immediate surgical intervention. In England, there are an estimated 600,000 emergency admissions under the care of general surgeons annually [2]. The assessment and management of these patients is a complex undertaking, requiring a holistic approach incorporating clinical examination, biochemical parameters, and radiological imaging.

Cross-sectional imaging in the form of contrast-enhanced abdominopelvic computed tomography (CT) remains the gold standard of investigation for most abdominal pathologies [3]. In practice, there are relatively few occasions in which a patient cannot be sufficiently stabilized prior to undergoing preoperative imaging, as the information obtained from these scans in terms of diagnosis and impact on surgical management is invaluable. Given the potential risks, morbidity, and mortality associated with emergency surgical interventions, preoperative imaging remains a crucial diagnostic tool. The 2011 report, "The Higher Risk General Surgical Patient" [4], published by the Royal College of Surgeons, detailed emergency surgery as a high-risk and high-cost area of acute surgical care, on a previously underestimated scale. Patients belonging to this category often have high postoperative critical care demands and, in many instances, spend prolonged time in intensive or high-dependency surgical units postoperatively [5]. In the 2010 Intensive Care National Audit and Research Centre report, surgical emergency cases alone accounted for 14,000 admissions to intensive care in England and Wales annually, associated with an estimated cost of £88 million [6]. A cost-effective analysis study conducted at a single center in the United Kingdom estimated the total inpatient cost of emergency laparotomies within their surgical unit at £5 million per annum (£4,992,572), equivalent to £13,000 per patient. When these figures are applied nationally, the annual inpatient cost of emergency laparotomies to the National Health Service in England is estimated at £650 million [7].

The impetus for this study was the recognition of an uncharacteristically high number of negative laparotomies within our general surgery department and on-table, intraoperative findings that contradicted preoperative radiology reports. For context, Ealing Hospital is a busy district general hospital serving a catchment area of over 300,000 patients [8]. Our radiology department consists of a team of in-house substantive radiology consultants who report scans during the daytime. Out of hours, however, to meet the demand for reporting of emergency scans, our hospital relies on a teleradiology system: Medica (Medica Group, England, UK), where the scans are vetted and reported by off-site radiologists.

The interpretation of emergency abdominopelvic CT imaging performed on acutely ill patients is complex [9]. Despite being both highly accurate and sensitive at identifying most intra-abdominal pathologies [9,10], the implications of inaccurate reports in the context of an acutely unwell surgical patient can be disastrous.

At present, the Royal College of Radiologists has not publicized guidance on the accepted standard of discrepancy rates for cross-sectional imaging. In 2017, Howlett et al. [11] published recommendations for audit standards following a comprehensive literature review. The target major discrepancy rates for reports generated by consultant radiologists both off and on-site were proposed at <5%.

The primary aim of this retrospective observational study was to assess the discrepancy rates for patients undergoing abdominopelvic CT for acute non-traumatic abdominal pain who subsequently undergo emergency surgery at our surgical unit. Emphasis was placed on identifying the differences between preoperative radiology reports, on-table intraoperative findings, and the source of the report, thus allowing us to identify factors that may contribute to the discrepancies, generate learning points, and provide recommendations to inform current practice.

## Materials and methods

Study design and selection of participants

This retrospective single-center observational study was conducted in the General Surgery department of Ealing Hospital, London North West University NHS Trust from August 2022 to February 2023 inclusive. All adult patients aged 16 years and over presenting to accident and emergency with atraumatic abdominal pain who underwent abdominopelvic CT imaging with subsequent surgical intervention (laparoscopy and or laparotomy) during the stipulated study period were included. Patients whose operative intervention was delayed more than 24 hours from issuance of their final radiological report were excluded from the study.

Data collection

Patient demographic data, radiology reports, and operative notes were sourced from electronic notes. Additional data was collected on the source of the radiological reports, either from in-house radiologists or those outsourced to the teleradiology company Medica. Surgical data were extracted from the hospital’s electronic operating theater planner (Newton) and surgical operative notes. Patient demographic data, time elapsed between the provision of the final radiological report, and operative start times were recorded. Radiological reports were extracted from our online reporting database, picture archiving and communication system. The primary radiological conclusion or diagnosis, issuance time of the final report, and the source of the report (in-house radiologists versus outsourced radiologists) were documented. All data were entered manually and collated into Excel spreadsheets (Microsoft Corporation, Redmond, WA, USA).

Discrepancy coding

Two senior surgeons independently compared the preoperative radiology reports and intraoperative findings to identify any discrepancies; they were both in agreement. Although there is little consensus in the published literature of what constitutes a "major" versus a "minor" discrepancy, for this study, we have limited the data collection to major discrepancies, defined as a CT report finding or lack thereof, which resulted in the incorrect surgical management of the patient in such a way as to cause patient harm [12]. Discrepancy rates were calculated as the ratio of the number of discrepancy cases to the total number of cases for that group.

Study approval

This was a retrospective single-center observational study comparing the hospital’s clinical service outcomes against nationally agreed targets. The study was registered, and ethical approval was obtained from the NHS Foundation Trust Clinical Governance Department (approval number: SUR.EH.22.334). The requirement for patient consent was waived given the retrospective nature of the study, and formal ethical approval was not required as per the UK National Research Ethics Service guidelines.

Statistical analysis

Descriptive statistics are reported as means and standard deviation, median with interquartile range (IQR), or as otherwise indicated. The frequency of all major discrepancies was calculated with comparisons made between the in-house and out-of-hours (Medica) radiologists. Fisher’s exact test was used to calculate the statistical significance of the discrepancy rates in the two groups. All analysis was undertaken using PRISM 10 statistical software (GraphPad Software, La Jolla, CA, USA).

## Results

**Figure 1:**
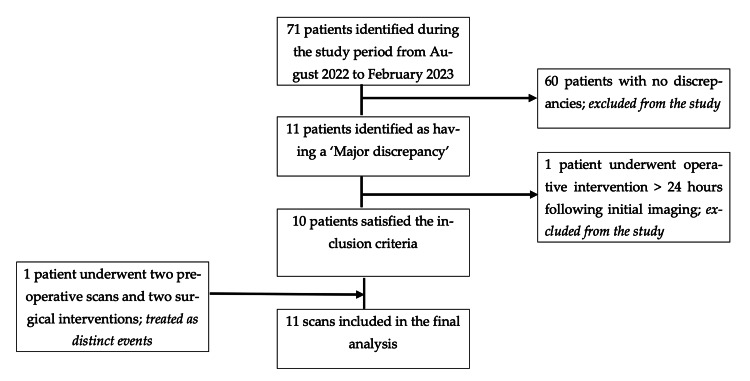
Study population

During the study period, 71 acute presentations were recorded. For 60 patients, the intraoperative findings were consistent with their preoperative radiological reports and, as such, were excluded from the study. Of the remaining 11 patients with major discrepancies identified, one patient underwent operative intervention at a time greater than 24 hours following their initial CT report and was discarded from the study participants. One patient underwent two separate preoperative scans and two distinct surgical interventions. For the purposes of data analysis, these were treated as discrete events. An overview of the enrolment process is outlined in Figure 1.

Demographic data

**Figure 2:**
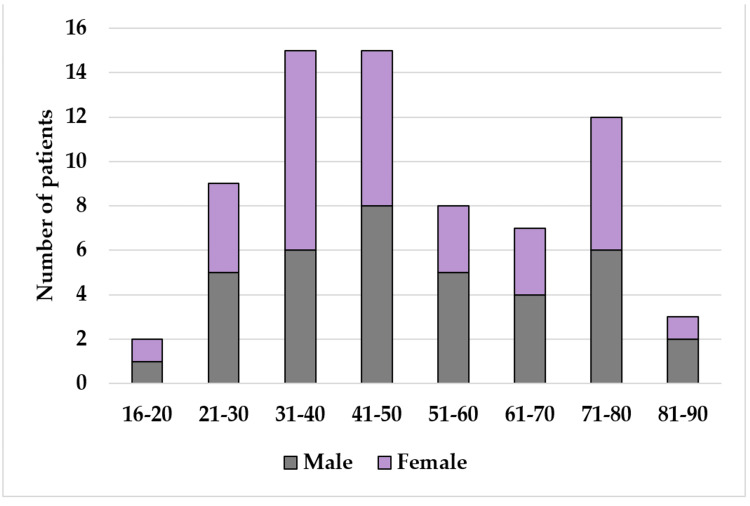
Bar chart depicting the distribution of patients by age range for all patients identified (n=71)

The average age of the patients was 45.1 years (range 20 to 73, median 45.5, IQR 12). The proportion of operated cases of non-traumatic acute abdomen was significantly higher in males (70%) as compared to females (30%). See Table 1 and Figure 2 for a comprehensive breakdown of all patients identified.

**Table 1:** Patient demographic data

Variables	Sex		N	Sex		N
	All (n=71)			Study participants (n=10)		
Age range	M	F		M	F	
16-20	1	1	2	0	1	1
21-30	5	4	9	1	0	1
31-40	6	9	15	0	1	1
41-50	8	7	16	4	1	5
51-60	5	3	8	0	0	0
61-70	4	3	7	1	0	1
71-80	6	6	11	1	0	1
81-90	2	1	3	0	0	0
Total	37 (52.1%)	34 (47.9%)	71	7 (70%)	3 (30%)	10

Discrepancy rates

**Figure 3:**
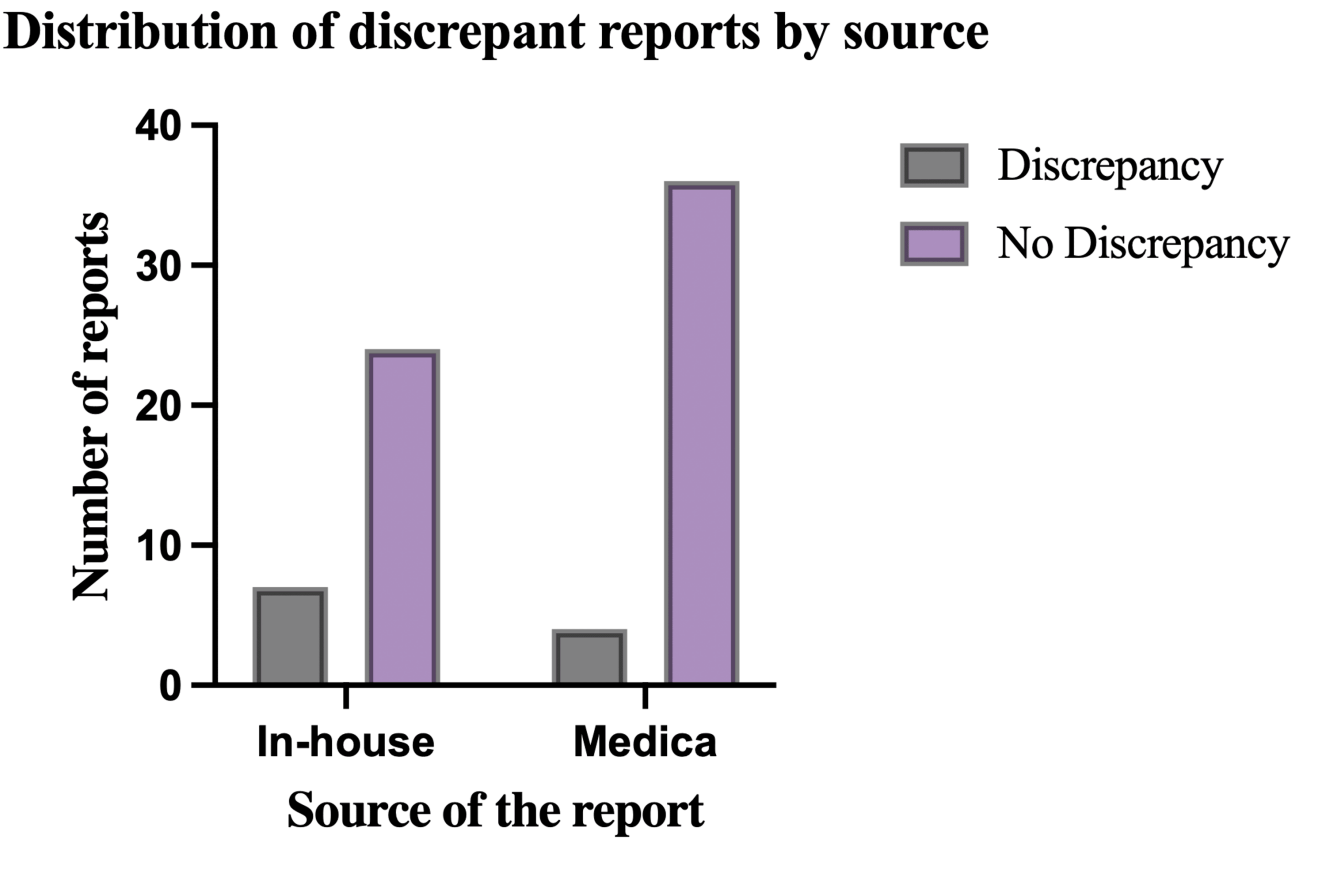
Bar chart representation of the number of discrepant reports by source

Eleven of the 71 scans were found to have a major discrepancy between radiological reports and intraoperative findings, corresponding to an overall discrepancy rate of 15.5%. Seven of the 11 reports were issued by our in-house radiologists (63%), and four by the teleradiology team (36%). Overall discrepancy rates were calculated at 4/71 (5.6%) for Medica and (7/71) 9.9% for reports generated by the in-house radiologists (Table 2, Figure 3).

**Table 2:** Contingency table for discrepant reports

Reporting source	Major discrepancy		Total
	Yes	No	
In-house	7	24	31
Medica	4	36	40

Description of discrepancies

Four of the 10 patients had radiological diagnoses of acute appendicitis, one of which had a macroscopically normal-appearing appendix with hemoperitoneum discovered within the pelvis and cystic structures identified within the pouch of Douglas. A second patient’s preoperative scan reported no features of acute appendicitis; however, intraoperative findings revealed a perforated appendix with four quadrant pus, omental adhesions, and inter-loop abscesses. Three scans reported varying degrees of bowel obstruction; one scan noted a transition point in the right upper quadrant, and the other two commented on intussusception as a possible etiology. One report noted a perforated duodenal ulcer; however, intraoperatively, a 2 mm jejunal ulcer was identified. The final two scans (performed on the same patient) reported a poorly enhanced mid-ileal loop with mesenteric stranding, suspicious for ischemia on the first report. A second report, performed five days following the first, identified a grossly dilated small bowel with air locules in the dependent portion of the left bowel, reiterating concerns for bowel ischemia. In both instances, the on-table intraoperative findings differed from those reported on the preoperative scans. An overview of a random sampling of these findings is outlined in Table 3.

**Table 3:** Random sampling of discrepancies identified

Radiological report	Intraoperative findings
Peri-appendiceal inflammatory changes, appendix normal size, early mild acute appendicitis	Hemoperitoneum in the pelvis with a cyst in the pouch of Douglas, appendix normal
Acute retrocecal appendicitis. No evidence of localized perforation or collection	Inflamed appendix. Blood in the pelvis and left paracolic gutter. Endometriotic patches in the pelvis, both paracolic gutters and on the diaphragm above the liver
Acute appendicitis associated with appendicoliths and peri-appendiceal fatty stranding	Inflamed retrocecal appendix adherent to posterior wall of caecum. The middle of the appendix had eroded into the posterior wall of the cecum, with perforation at both the middle of the appendix and the posterior caecal wall
Uncomplicated acute appendicitis	Gangrenous perforated appendix, adherent to the pelvis, three quadrant peritonitis, pus on the small bowel
Either acute small bowel obstruction secondary to adhesions/congenital bands or an intussusception secondary to an intraluminal polypoid mass in the terminal ileum is suspected	No intussusception. Small bowel examined from duodenojejunal flexure to terminal ileum. Distended small bowel with intermittent collapsed loops but no transition point. Terminal ileum and caecum appeared normal

## Discussion

Despite significant improvements in clinical investigations and advancements in diagnostic methods, accurate diagnosis of an acute abdomen remains a difficult undertaking [13]. The successful management of these patients relies on differentiating between those requiring operative intervention and those who do not, with the decision to operate relying on careful correlation between objective clinical signs, biochemical parameters, and radiological findings. The importance of accurate preoperative diagnosis cannot be understated, as the morbidity and mortality associated with unnecessary surgery are significant [14].

Inaccurate radiological reports can lead to delays in diagnosis, overinvestigation, or inaccurate management altogether. The primary parameter by which the accuracy of these reports is assessed is the "discrepancy rate," whereby a discrepancy is defined as the existence of differences between the CT report and the operative findings [11]. The Royal College of Surgeons, High-Risk General Surgical Patient 2011 report defines a "significant discrepancy" as an error of fact in the radiology report, which leads to incorrect management or patient harm, and is determined by a multidisciplinary review of the imaging report, imaging findings, and operative findings [4].

In the United Kingdom, to meet the rising demand for the number of urgent and emergency scans requested out-of-hours, many hospitals have outsourced their out-of-hours radiology services to teleradiology companies. Current literature addressing this topic has identified sub-specialty reporting and the clinical seniority of the reporter as potential contributing factors. However, there is a relative paucity of literature describing discrepancy rates by report source (in-house compared to outsourced).

Our study revealed that the discrepancy rates of our onsite radiologists exceeded those of the outsourced reporting services, 9.9% and 5.6%, respectively. The discrepancy rates of both our in-house and outsourced radiology reports were higher than the national standard of <5%; however, this did not achieve statistical significance. This contradicts the results of similar studies, where the lowest discrepancy rates were demonstrated when on-site consultant radiologists reported scans [15]. Ours is the only study to our knowledge demonstrating greater discrepancy rates by in-house radiologists compared to outsourced reports.

In the above definition of a "significant discrepancy," emphasis is placed on two primary outcomes: incorrect management and patient harm. When reviewing our discrepancy data, it can be argued that many of the patients (for instance, those in whom appendicitis was the primary radiological diagnosis) underwent the same final surgical management; however, the authors believe that in many of these cases, the severity of the findings was either underreported or entirely inaccurate, potentially affecting the NCEPOD category selected and ultimate time or urgency to theatre. Furthermore, in such cases where the predominant pathology was gynecological, accurate preoperative radiological reporting would lend itself to better perioperative planning, for instance, a joint surgical-gynecological approach rather than having to prolong patient anesthetic time to facilitate an on-table specialty review.

An important exclusion criterion for our study was patients with a scan-to-theater interval that exceeded 24 hours, as we appreciate that within this time, the patient’s clinical condition may have progressed, potentially contributing to the discrepancy in severity between the scan and table findings. However, this theory only addresses the difference in the severity of findings, which relies on the assumption that the original diagnosis was correct. Our findings revealed that in many instances, the initial radiological diagnosis varied significantly from the intraoperative findings (Table 3).

The etiology of discrepancy rates for cross-sectional abdominal imaging is undoubtedly complex and multifactorial, and the accuracy of radiological reports is dependent on a multitude of factors, both technical and clinical. One potential solution to improving this may lie in the double-reading of CT reports for patients undergoing surgical interventions. One study examining abdominopelvic CT in surgical patients reported a 14% rate of management changes following double reading of initial reports [16]. Notably, in the emergency or out-of-hours setting, the authors appreciate that this may not always be feasible.

A second suggestion to improve the observed discrepancy rates involves fostering greater communication and interdisciplinary collaboration between surgeons and radiologists. Multiple studies have evaluated the significance and implications of insufficient or incomplete clinical details on radiological requests and emphasized the importance of clear communication of the pertinent patient history and clinical questions to the reporting radiologist [17,18].

Advances in modern medicine have created an almost sterile communication environment between requestor and reporter, with electronic requests and dictated radiology reports adding yet another element of ambiguity [19]. One North American study explored this link, reporting the potential implications of in-person communication between surgeons and radiologists on surgical decision-making. In-person collaborations led to changes in surgeons’ diagnostic impressions in 43% (43 of 100) of cases and an equivalent change (43%) in the medical and/or surgical planning of these cases [20]. Thus, highlighting that improved communication between surgeons and radiologists has the potential to significantly impact patient care and surgical outcomes.

Study limitations

The primary limitation of this study was its retrospective design compounded by the selection of patients from a single department within a single center, lowering the level of evidence and external validity of the study. The findings of the study are reliant on the accuracy and reliability of retrospectively collected data, and the study design precluded sample size calculations as all patients who met the inclusion criteria during the study period were enrolled retrospectively. As a result, the sample size may not have been adequate to demonstrate statistical significance for discrepancy rates. It is possible, however, that with a larger sample size, the trend in the discrepancy rate demonstrated may achieve statistical significance. Further, although we excluded patients with a scan to operative start time exceeding 24 hours, this figure was arbitrarily selected, and the progression of disease severity from the time of imaging to operative start times is entirely unpredictable. A final limitation would be the subjective nature of the interpretation of on-table findings and macroscopic pathology. To counteract this, two senior surgeons independently decided which cases were deemed to have "major" discrepancies.

## Conclusions

Interpretation of abdominal pain remains a challenging undertaking for surgeons. Accurate diagnosis relies on the correlation between clinical, biochemical, and radiological findings. Abdominopelvic CT remains the gold standard for identifying most intra-abdominal surgical pathology, yet the interpretation of these images in the context of an acutely unwell surgical patient is challenging. The morbidity and mortality of emergency abdominal surgery are high, and as such, the significance of accurate reports cannot be understated. Our study revealed discrepancy rates above the national standard (<5%) with no statistical significance in the discrepancy rate depending on the source of the report (in-house compared to outsourced). There are a plethora of potentially complex and multifactorial reasons driving these discrepancies; ultimately, a multidisciplinary, cross-specialty approach is required to address these issues within our department and improve patient care.
